# Optimizing transcranial magnetic stimulation for spaceflight applications

**DOI:** 10.1038/s41526-023-00249-4

**Published:** 2023-03-28

**Authors:** S. M. Romanella, L. Mencarelli, K. Seyedmadani, S. Jillings, E. Tomilovskaya, I. Rukavishnikov, G. Sprugnoli, S. Rossi, F. L. Wuyts, E. Santarnecchi

**Affiliations:** 1 Precision Neuroscience and Neuromodulation Program, Gordon Center for Medical Imaging, Radiology Department, Massachusetts General Hospital, Harvard Medical School, Boston, MA USA; 2 Non-invasive Brain Stimulation Unit, IRCSS “Santa Lucia” Foundation, Rome, Italy; 3 Biomedical Engineering Department, University of Houston, NASA Johnson Space Center Houston, Houston, TX USA; 4 Lab for Equilibrium Investigations and Aerospace (LEIA), University of Antwerp, Antwerp, Belgium; 5 Institute of Biomedical Problems, Russian Academy of Sciences, Moscow, Russia; 6 Siena Brain Investigation & Neuromodulation Lab (Si-BIN Lab), Department of Medicine, Surgery and Neuroscience, Neurology and Clinical Neurophysiology Section, University of Siena, Siena, Italy; 7 Human Physiology Section, Department of Medicine, Surgery, and Neuroscience, University of Siena, Siena, Italy

**Keywords:** Neuroscience, Biomarkers

## Abstract

As space agencies aim to reach and build installations on Mars, the crews will face longer exposure to extreme environments that may compromise their health and performance. Transcranial magnetic stimulation (TMS) is a painless non-invasive brain stimulation technique that could support space exploration in multiple ways. However, changes in brain morphology previously observed after long-term space missions may impact the efficacy of this intervention. We investigated how to optimize TMS for spaceflight-associated brain changes. Magnetic resonance imaging T1-weighted scans were collected from 15 Roscosmos cosmonauts and 14 non-flyer participants before, after 6 months on the International Space Station, and at a 7-month follow-up. Using biophysical modeling, we show that TMS generates different modeled responses in specific brain regions after spaceflight in cosmonauts compared to the control group. Differences are related to spaceflight-induced structural brain changes, such as those impacting cerebrospinal fluid volume and distribution. We suggest solutions to individualize TMS to enhance its efficacy and precision for potential applications in long-duration space missions.

## Introduction

As space agencies prepare for longer space missions to reach and colonize Mars, crew-members’ health and operational performance have become a crucial concern. Space travelers undergo multiple classes of stressors, as recognized by the Human Research Program (HRP) roadmap^1^, including exposure to unnatural gravity fields and cosmic radiations, and the consequences of living in a confined and isolated environment. Therefore, the need to evaluate acute and long-lasting health consequences of space traveling and develop new potential countermeasures arises. In this framework, non-invasive brain stimulation (NIBS) could provide a useful tool in space exploration.

NIBS encompasses multiple techniques capable of modifying brain activity via the transcranial delivery of magnetic pulses or electric currents. Among them, transcranial magnetic stimulation’s (TMS) various protocols may represent a valid set of countermeasures for a wide range of risks associated with spaceflight^2^. TMS is based on Faraday’s principle of electromagnetic induction: a pulse of electrical current flows through loops of wire (forming the magnetic coil) and generates a rapidly changing magnetic field perpendicular to the coil plane, that induces an electric field parallel to the inner surface of the conductor. When delivered close to the individual threshold of stimulation, the induced electric field depolarizes the dendrites of pyramidal neurons trans-synaptically^3^. Electric fields of different strengths and forms can be generated by the TMS stimulator through the modification of physical parameters, such as magnetic pulse waveform, coil shape, orientation, as well as intensity and frequency of stimulation^4^. The safety and efficacy of TMS has resulted in its approval by the Food and Drug Administration (FDA) for the treatment of drug-resistant depression, obsessive-compulsive disorder, migraine, and smoking addiction^5^. Other neuropsychiatric disorders may also benefit from specific TMS protocols that can induce long-lasting changes in the synaptic plasticity of the target region and connected networks in a controllable manner^6,7^.

In the domain of space exploration, TMS could be a useful tool before, during, and after space missions. It may be used to support the psychological well-being and cognitive performance of crew-members both on the International Space Station (ISS), planetary surface installations, and after spaceflights^2^. TMS may also be deployed as a purely research-oriented tool, to investigate the brain’s response to space missions and define biomarkers of this adaptation, by collecting data on cortical excitability, neuroplasticity, and brain connectivity levels^8–10^. However, individual differences in brain morphology can highly impact TMS efficacy and they need to be carefully considered before any practical applications^11^. This is particularly relevant due to the functional and structural modifications that astronauts undergo during space missions (for a detailed review see ref. ^12^). Indeed, space stressors seem to induce changes in brain anatomy, particularly in cerebrospinal fluid (CSF) volume and distribution, gray matter shape change, and local skull-to-brain distance, due to the upward shifting of the brain^12^. This issue can be tackled by predicting the distribution of the electric field following the TMS pulse using individual neuroimaging data, such as structural magnetic resonance imaging (MRI). Software running biophysical modeling algorithms have been developed to create realistic computational models of the head and run simulations of TMS administration. Individual anatomical MRI images are segmented into major tissue classes, and a 3D volume conductor model of the head is created by surface reconstruction with specific intrinsic tissue conductivities. After setting the parameters and specifications of the stimulation (e.g., coil type, position and orientation, intensity of stimulation), the software will compute the propagation of the induced electric field in the head. The output is a heat map of the electric field induced by the TMS pulse in the individual 3D head. Because the electric field is a vector field, it can be represented by NormE, a scalar parameter representing the strength of the vector, irrespective of its direction. The key factor of these models is a precise description of the geometry of the head as a volume conductor, assuming the tissues’ electric properties with a finite element method (FEM) model^13,14^. Using individualized models provides better control of the electric field to achieve greater efficacy in any TMS application.

In this context, the present work aims to optimize TMS for space exploration, taking into consideration potential spaceflight-associated brain changes. MRI T1-weighted scans of 15 Roscosmos cosmonauts were collected before, immediately after, and 7 months after a space mission lasted an average of 6 months on the ISS. MRI data from 14 non-flyer controls were collected at the same time-points. We simulated the electric field (represented as NormE) generated by TMS over different brain regions associated with primary cognitive and motor functions. To investigate specific changes in the modeled current strength generated by TMS over the target areas, the change in NormE over time was compared between cosmonauts and controls. Differences in the total CSF volume were also analyzed. We then offer two practical solutions for TMS personalization to increase its potential beneficial effects and provide a starting point for TMS applications on space missions, hoping to boost interest and effort to implement non-invasive brain stimulation techniques for space exploration.

## Results

### Spaceflight-associated changes

**Fig. 1:**
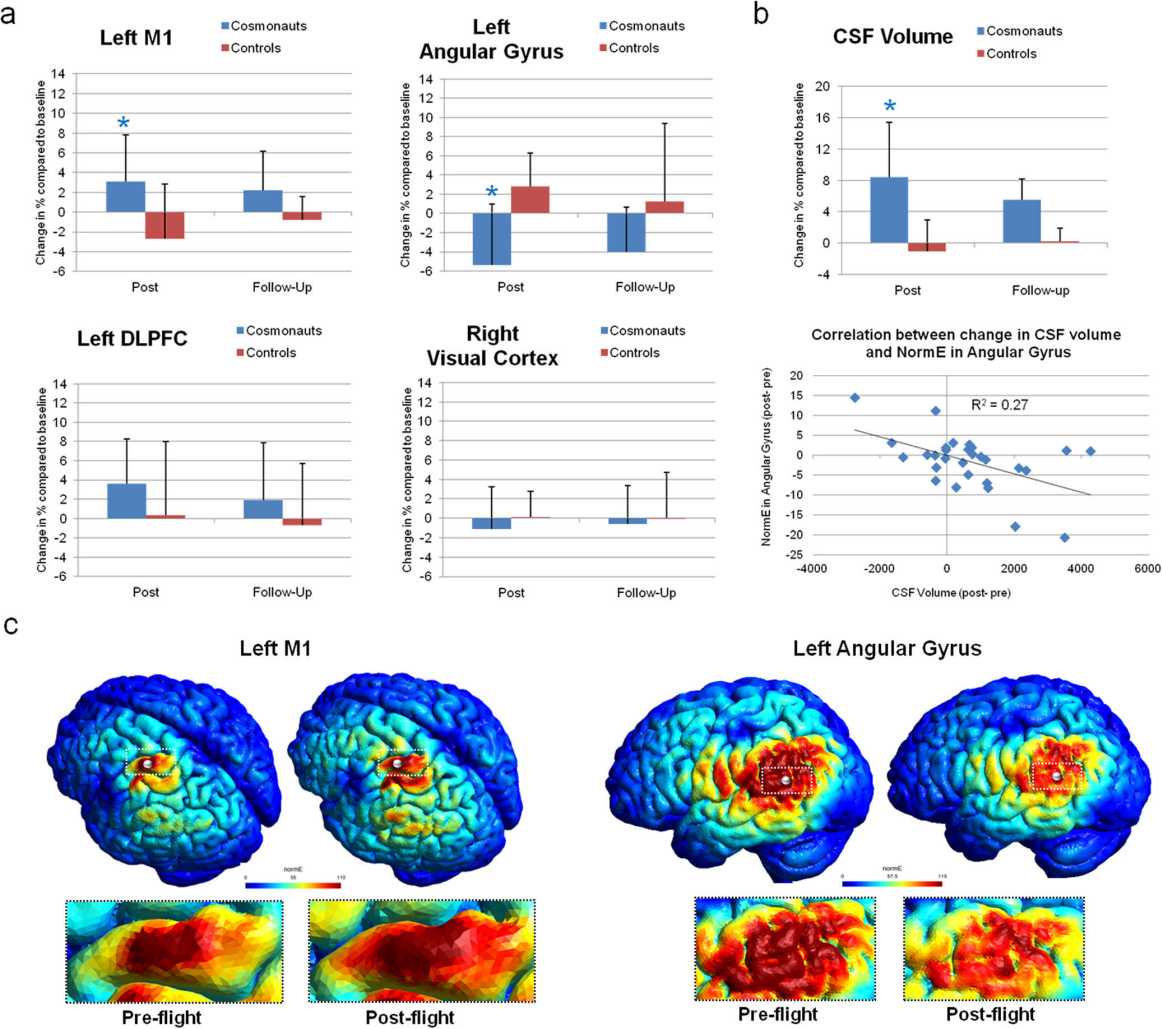
Changes in NormE and brain structure in cosmonauts. **a** The figure reports changes in NormE reaching the cortical regions in cosmonauts (blue bars) compared to non-flyer controls (red bars). Graphs show the current strength of the E-field expressed in % difference of NormE from the baseline (pre-flight) in the target area for all cosmonauts/controls on each time-point. We found statistically significant differences in NormE under the stimulation sites in post-flight compared to baseline in left M1 and left angular gyrus. Asterisks indicate significant differences between cosmonauts and controls. We provided error bars for each measure. **b** Differences pre vs. post-flight in total CSF volume in cosmonauts compared to controls are also reported (left). Finally, the correlation between the change in total CSF volume and the decrease in NormE reached in the gray matter in left angular gyrus in cosmonauts is displayed (*R*^2^ = 0.27, *p* = 0.004; right). The CSF volume is presented in mm^3^; while the NormE values are in V/m. **c** Below the graphs, we present pre-flight and post-flight electric field maps (NormE) reaching left M1 and the left angular gyrus (presented in V/m) induced by TMS pulse in one cosmonaut. Note the difference in electric field strength as represented in NormE over the interested area. The white spheres represent the cortical ROI used to extract the NormE values.

Our results showed significant differences in NormE under some of the stimulation sites post-flight in cosmonauts compared to controls (see Fig. 1). Specifically, cosmonauts presented a significant increase in intensity at post-flight when the stimulation site was left M1 [*F*_(1,27)_ = 4.28, *p* = 0.048]. In this group we also reported a statistically significant decrease in NormE when the stimulation was over the left angular gyrus [*F*_(1,27)_ = 10.37, *p* = 0.003]. No significant differences in NormE were found at post-flight under the left DLPFC and right visual cortex (both *p*-values > 0.05). When comparing cosmonauts and controls at follow-up vs. pre-flight and follow-up vs. post-flight, the two groups showed no differences in NormE in any of the stimulation sites (all *p*-values > 0.05). We also analyzed TMS-induced NormE in every area in the control group alone. No changes over time in NormE computed in any region were found (*p*-values > 0.05), suggesting that statistical differences in cosmonauts and controls seemed to be related to the time spent in space rather than variability in the cohorts. Total CSF volume change between baseline and post-flight was significantly higher in cosmonauts compared to controls [*F*_(1,27)_ = 17.39, *p* < 0.001]. The ANOVA on CSF pre-flight vs. follow-up and follow-up vs. post-flight showed no significant difference (*p* > 0.05).

The analysis revealed a significant negative correlation between the change in total CSF volume (post-flight – pre-flight) and the decrease of NormE reaching the left angular gyrus [*R*^2^ = 0.27, *p* = 0.004]. The analysis showed no relationship between CSF changes and NormE differences in M1, DLPFC, and visual cortex (all *p*-values > 0.05).

### TMS personalization

**Fig. 2:**
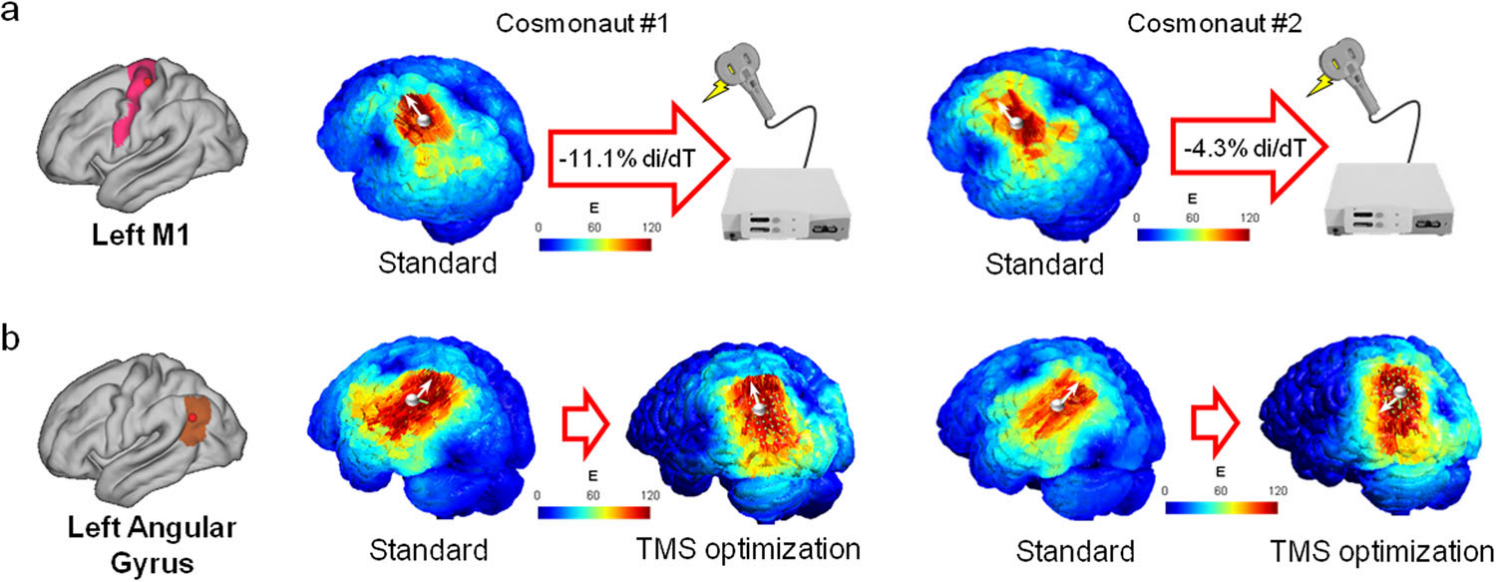
TMS optimization solutions. The figure shows potential solutions to individualize TMS parameters in two cosmonauts (center and right). **a** For stimulation over left M1, we lowered the intensity of TMS pulse by pre to post-flight difference in E-field. **b** Over the left angular gyrus, instead, we performed TMS optimization to find the best-individualized setting. The TMS optimization algorithm suggested a different coil position and orientation for each cosmonaut in order to induce the largest NormE (Cosmonaut #1: coil position *[x* = *–70.48, y* = *–74.28, z* = *29.95]*; coil orientation *[x* = *-–72.1, y* = *–64.63, z* = *42.71]*; Cosmonaut #2: coil position *[x* = *–70.72, y* = *–67.42, z* = *43.03]*, coil orientation *[x* = *–76.07, y* = *–59.86, z* = *34.13]*. The output includes the position and orientation of the coil that induces the largest NormE (white arrow indicates the direction of the coil). We show the different current distributions resulting from the standard simulation and TMS optimization. Values are in V/m. The white spheres represent the cortical ROI used to extract the NormE values. Results in NormE can be seen in Table 1.

We offer two potential solutions to ensure a more efficient TMS intervention (see Fig. 2). As aforementioned, compared to controls, cosmonauts showed an increase of NormE in left M1 and a decrease in left angular gyrus after the spaceflight. Therefore, we provide solutions to optimize stimulation for these two target sites. For stimulation over M1, we lowered the intensity proportionally to the post-flight change in NormE to reach levels similar to baseline. We ran a new simulation on the post-flight scan using the adjusted intensity values and found the NormE values to reach baseline levels (see Table 1, left). On the other hand, the decline in NormE over the left angular gyrus in post-flight might affect TMS efficacy. We ran a TMS optimization algorithm and reported the resulting NormE values compared to the previous standard simulation (see Table 1, right). The TMS optimization suggested us the best coil position and orientation to achieve stronger induced NormE in the left angular gyrus. For more information on the optimization procedures see the Methods section.

**Table 1:** NormE values resulted from standard and personalized stimulations.

	Left primary motor cortex			Left angular gyrus		
	Baseline	Post-flight		Baseline	Post-flight	
	Standard	Standard	Personalized	Standard	Standard	Personalized
Cosmonaut #1	71.60	79.98	70.61	79.08	71.94	80.16
Cosmonaut #2	63.69	66.44	63.59	77.41	64.79	78.58

We show the average NormE reaching left M1 and left angular gyrus in simulations ran from neuroimaging data collected in two cosmonauts. Values are extracted from standard simulations vs. personalized solutions and presented in V/m.

## Discussion

We analyzed the electric field generated by TMS over four brain regions using MRI scans collected from crew-members and controls pre and post space missions, and at follow-up. When compared to the control group, the cosmonauts exhibited significant differences in the modeled TMS-generated current after spaceflight. We demonstrate that flight-induced alterations in brain morphometry are partially responsible for these findings, pointing their relevance for determining the safety and efficacy of TMS stimulation. We introduce two practical solutions based on individual neuroimaging data and simulation to personalize TMS intervention for space travelers. Finally, we discuss the potential implementations of TMS in space missions as well as the limitations of the study.

After the space mission, the cosmonauts showed a significant increase in the current strength over the left M1 and a decrease in current reaching the angular gyrus. The analysis showed no statistical differences in the control group alone, suggesting that changes in NormE were associated with time spent in space and not variability within the groups. At the follow-up, cosmonauts presented a return of all measurements toward the baseline level, suggesting Earth-based re-adaptation. Differences in TMS-induced electric fields in space travelers can be caused by a plethora of various functional and structural cortical changes. One of the most important is CSF volume and redistribution^15^. In our study, space travelers showed an increase in total CSF volume after spaceflight, similar to previous studies^16–19^. This is a well-known phenomenon and one of the most recognizable biomarkers of spaceflight. It is also further relevant in brain stimulation studies, as TMS-induced current preferentially follows the path of least resistance, meaning that electric fields propagate more through CSF. The conductivity of CSF is considerably greater than the conductivity of any other brain tissue (by a factor of 15–30 at 4 kHz^20^. CSF redistribution will, therefore, affect the location of the peak TMS-induced electric field. Our study seems to confirm this association, as explained by the significant increase in total CSF volume accounting for over 25% of the variance in NormE in angular gyrus in cosmonauts.

Previous literature shows how changes in CSF are associated with an upward shift of the brain, consisting of a redistribution of the subarachnoid CSF that causes a reduced liquid volume at the vertex, coupled with an expansion at the bottom of the skull^16,21^. These findings have also been confirmed by studies using voxel-based morphometry (VBM)^21^ and free-water analysis of diffusion MRI (dMRI)^22^. However, high-induced electric fields are primarily found in gray matter regions adjacent to areas of reduced or thinning CSF thickness^15^. This would also decrease the distance between the brain and the coil. Therefore, an increase in the NormE in M1 following spaceflight may be explained by the presence of a thinner regional CSF layer on the vertex and the upward shift of the brain. On the other hand, there is no significant correlation between changes in total intracranial CSF volume and NormE over the M1. The electric field in M1 may be affected by the upward brain shift, not only because of CSF redistribution but also following cortical thickness and MRI diffusion alterations^15,16,21^. Therefore, it is not entirely surprising that we did not observe a significant correlation between specific, local E-field modifications and the total intracranial CSF volume.

The decrease in electric current reaching the angular gyrus seems to be influenced by an opposite modification in CSF volume. The literature reports an increase in CSF volume after spaceflight mostly affecting the skull base, Sylvian fissure, and temporal lobes^23^. The angular gyrus, which is close to the Sylvian fissure, is the most inferior brain target region we chose. The CSF layer close to this area has been repeatedly found to be increased after spaceflight^16,21^. This may explain the local decrease of NormE over the angular gyrus since a greater amount of CSF potentially causes widespread current shunting. We can speculate that the increase in total CSF volume explaining the variance in NormE in the angular gyrus may reflect the local increase in CSF. However, we did not assess the CSF variation on a region-by-region basis, therefore we cannot completely rule out the influence of other factors or confirm this hypothesis even if the present results are in line with the previous literature and proposed mechanisms. Finally, we did not observe significant variations in the NormE field simulated over the DLPFC and visual cortex at baseline vs. post-flight simulation. The present investigation did not aim to assess the morphovolumetric changes that affect the brain after spaceflight, so we cannot provide a definitive and certain explanation of the physiological process that may have influenced the tested brain regions and thus the electric field. Nonetheless, the findings could be related to the fact that these regions are less affected by the upward brain shift occurring after spaceflight and by significant CSF redistribution, as presented in recent literature^16,21^.

At follow-up, total CSF volume exhibited a re-adjustment toward the baseline level in cosmonauts (see Fig. 1). As follow-up MRI scans were collected a considerable time after re-entry (7 months), this may signal an homeostatic re-adaptation to Earth-based gravity, as previously observed and suggested in other studies^23^. However, the time required for the effects of spaceflight on brain structure to reverse completely is currently unknown, as further follow-ups (1 year, 2 years) have not yet been tested. Therefore, it is uncertain if the decrease toward baseline seen at ~7 months after re-entry was reaching the plateau or continuing in the process of re-adaptation. Longer space missions, as required for Mars colonization, may also exponentially accentuate these modifications to brain structure. A longer period of Earth-based re-adaptation may be needed to return to values close to the baseline. In this situation, TMS may be a potential tool for investigating the neural process triggered by spaceflight-associated adaptation. To overcome differences in morphology and volume due to spaceflight and consequent re-adaptation, a call for individualization is needed.

Considering the differences in NormE generated by TMS due to various concurring factors, great care must be taken when inducing electric fields at any stage of space missions. Spaceflight-induced modifications in anatomy need to be considered, such as CSF volume and distribution, local skull-to-brain distance due to the upward shifting of the brain, and cortical thickness modifications. Along with a standard inter-individual variability, this may result in fundamental differences in the electromagnetic field exposure across subjects, even for an identical stimulation dose (for a review of the parameters and dose personalization, see ref. ^11^). Ignoring these modifications may result in various consequences, such as loss in the focality of the field. This is particularly relevant for the beneficial effect of TMS, which could fall under the threshold and consequently become inefficient. Furthermore, this argument must be addressed for its relevance in terms of safety. Structural brain changes may unexpectedly increase the induced current strength reached in target cortical areas. Therefore, developing models that can mimic the generated E-field with high accuracy is crucial for TMS feasibility and future implementations in space missions.

Following this framework, we can personalize the setting by adjusting the parameters of the TMS pulse, correcting for changes in brain structures. The induced electric field distribution also depends on multiple stimulation parameters, such as the location and orientation of the TMS coil. In this study, two potential solutions for providing a personalized TMS application have been presented. The first involves adjusting the intensity of the TMS pulse (through the di/dT of the simulation parameters) to match the different current strengths reaching the relevant area. The TMS optimization algorithm, instead, compares a large number of potential coil positions and orientations to find the setting that generates the strongest E-field in the target area. Furthermore, while using T1-w MRI provides valuable input for the creation of a model, recent literature has also suggested diffusion MRI as a tool to gather data for biophysical modeling^24^. These techniques estimate tissue conductivities and can refine the precision of computational simulations.

A call for individualization is pivotal for exploiting the opportunities TMS can provide in the various stages of space missions (for a detailed review of the subject and potential challenges, see ref. ^2^). We briefly discuss two potential practical implementations: (i) TMS-EEG in different areas to investigate perturbation-based biomarkers associated with spaceflight, and (ii) repetitive TMS (rTMS) over the left DLPFC to mitigate challenges in operational performance and potential mood changes in-flight.

First, TMS may be a useful tool for investigating differences in cortical excitability before and after missions as well as on Earth-based space analogs. A few studies implemented a single-pulse TMS to investigate corticospinal excitability in hypergravity and microgravity. Davey and coworkers (2004) administered TMS over M1 on three healthy subjects during 10 parabolic flights to produce and record MEP in the deltoid and left and right erector spinae (ES) muscles. The data showed a similar pattern in all participants, revealing the facilitation of MEP responses in left and right ES muscles in periods of zero gravity (0 G). The MEP’s increase suggested that microgravity induced the activation of ES muscles through an increase in corticospinal excitability^25^. This result was also corroborated by studies on head-down-tilt bed rest (HDBR^26^). According to the authors, TMS can be used as a possible countermeasure against lower extremity dysfunction. TMS may fit into a countermeasure regime on long-duration space missions to counteract the effect of microgravity or for functional recovery after injury^27^. However, a protocol involving TMS combined with electroencephalography (EEG) may help identify perturbation-based biomarkers associated with spaceflight. By controlling the input to the cortical areas (i.e., the TMS pulse) and recording brain responses through EEG (TMS-EEG), we can quantify the transmission of generated activity as well as local response, propagation speed, and the dynamic spatial spreading of the current. TMS-EEG investigates causal communication between brain connections with a high temporal resolution, providing insights into mechanisms of effective connectivity^28^. A protocol of test-retest performed pre- and post-flight could be easily implemented as part of routine health screenings and follow-up visits. Furthermore, a TMS-EEG protocol for space travelers would identify predictive space-associated biomarkers of changes in local plasticity, cortical excitability, connectivity, and changes in induced brain oscillations.

On the other side, TMS may promote the psychological well-being and cognitive performance of crew-members during and after space missions. A recent study investigated the consequences of prolonged space exposure on two identical twins—one of whom spent a year on the ISS^29^. A post-flight decrease in cognitive speed and accuracy was observed in the sibling who went to space; this persisted up to 6 months after the end of the mission^29^. This post-flight cognitive deficit also involved signs of altered mood and anxiety. TMS may be implemented with cosmonauts in case of depressive episodes. Prefrontal daily rTMS over 4 to 6 weeks (20 to 30 sessions) has been approved by the FDA for the treatment of major depressive disorder (MDD) in adults^30^. The efficacy and safety of rTMS on the left prefrontal region was confirmed in two large, multisite, randomized controlled trials^31,32^ and one multisite trial that used a form of more focalized TMS (DeepTMS^33^). A cycle of rTMS may be easily implemented to optimize compensatory strategies and support cognitive performance.

More importantly, TMS treatment for depression or cognitive enhancement during space missions should be considered in-depth and explored further. While TMS may seem impractical on the ISS or spaceflights owing to its weight and interference with the magnetic field, it is worth noting that potential TMS application during a space mission has already been explored. Space environments, such as the ISS, spacecraft, the Moon, and Mars will necessitate more innovative solutions than Earth-based non-laboratory settings. Such environments differ from Earth in several aspects, including temperature and pressure^1^, and these may potentially alter the functionality of brain stimulation devices. This will hence require a TMS device capable of functioning outside laboratory settings and in the absence of trained operators. A recent study of an individually tailored TMS helmet applied over M1 suggested it to be a feasible and reliable alternative to traditional laboratory settings^34^. The same group tested the helmet at 0 G^35^. The investigators recorded the resting motor thresholds (rMTs), stimulating M1 with single-pulse TMS on 10 participants before, during, and after a parabolic flight. They showed how zero gravity motor thresholds were lower than Earth rMTs at the baseline. This reduction was recovered immediately post-flight, with a level similar to the pre-flight baseline. They ascribed this to the physical upward shift of the brain within the skull. A lower scalp-to-cortex distance would require an electric field less intense to induce the same cortical activation. This is in line with our increase in the current strength in M1 at post-flight as compared to the baseline. While more investigation is required, this has proved how TMS can be implemented in non-laboratory settings with variable gravity. This solution may create opportunities for in-flight TMS implementations to support crew-members in their daily work on the ISS and planetary surface installations.

Although we analyzed a unique longitudinal dataset of MRI data from cosmonauts matched with a control group, the study has a few limitations that should be discussed. First, our findings result from a computational model. Nonetheless, the analyses of the TMS-induced field and CSF in this study are based on biophysical modeling performed on real neuroimaging data collected on space travelers, a widely accepted procedure also in clinical settings. Additionally, previous literature confirmed that biophysical modeling is an accurate tool to simulate E-fields generated by TMS. The process of brain images segmentation, biophysical modeling, and NIBS simulations, indeed, has been successfully validated with real neurophysiological recordings and direct in-vivo intracranial measurements of the electric fields in previous extensive literature^36–41^. Likewise, other studies specifically validated the SimNIBS software by comparing the simulation output to collected real data^42,43^. Nevertheless, empirical data should still be collected in the future to confirm differences in TMS-induced electric fields due to morphological changes during space missions and provide new insight into the matter. Another limitation lies in the individual variability among space travelers due to previous training/education. Furthermore, the cosmonaut cohort contained a combination of first-time and experienced flyers. For space travelers who already performed a space mission, the data at pre-flight may deviate from the baseline level similar to all first-time flyers. Although this may confound space mission-induced effects, it also confirms the necessity of adjusting TMS settings to personalize interventions in this special cohort. Another confounding factor involves the timeline constraints regarding the acquisition of neuroimaging data, driven by logistic and organizational constraints. Post-flight MRIs were collected after an average of nine days upon returning to Earth. In our study, we showed that time spent after being back on Earth can partially counteract the spaceflight-associated changes in the brain. Although we do not think that the difference of a few days would be sufficient to trigger brain re-adaptation alone, we have to consider that earlier scanning session may reveal more pronounced effects of spaceflight on the brain. Similarly, the method section specifies the missing MRIs of space travelers whose scans we were not able to collect on follow-up, limiting the sample size at this time-point.

In conclusion, we demonstrated that the same TMS protocol generates different modeled current strengths in cortical targets after spaceflight as compared to pre-flight. These differences are partly due to spaceflight-induced changes in the CSF volume and distribution. We also discussed the use of individualized TMS applications in the different stages of space missions. Different TMS protocols can reveal specific biomarkers of brain adaptation to spaceflight and support crew performance and well-being. Personalizing TMS via biophysical modeling can overcome differences in brain morphometry due to physiological adaptation induced by space stressors. This may increase its specificity and further enhance its beneficial effects.

## Methods

### Subjects and data collection

A total of 29 male subjects were enrolled in the study: 15 cosmonauts and 14 matched controls. The two groups did not significantly differ for age. Three cosmonauts were first-time flyers, while the others had previously performed at least one space mission. Experienced flyers spent around 7 months prior in space. No participant took CNS-acting drugs during the time of the study. All subjects provided written informed consent before they participated in the study. The study was approved by the Institutional Review Board of Antwerp University Hospital (13/38/357), the Committee of Biomedicine Ethics of the Institute of Biomedical Problems of the Russian Academy of Sciences, and the Human Research Multilateral Review Board (HRMRB).

All cosmonauts were scanned at 3 time-points: ~3 months before launch (pre-flight), around 9 days after return from their 6 months duration spaceflight (post-flight), and on average 7 months after their return (follow-up). In the control group, there was an interval of 7 to 8 months between the first two scans and an interval of 16 months between the second and the third scans (see Table 2). When collecting data at follow-up, we had a smaller sample of space travelers and control participants due to the voluntary discontinuation of the study (final follow-up count for cosmonauts: *n* = 11 and controls: *n* = 8).

**Table 2:** Characteristics of the sample.

	Age	Prev mission	T1 (pre)	Mission	T2 (after)	Interval T1-T2	Interval T2-T3
	Years	Days	Days	Days	Days	Days	Days
Cosmonauts	46.06 (4.94) [39.8–56.47]	206.26 (182.3) [0–709]	86.5 (31.75) [27–147]	184.46 (48.98) [115–311]	9 (2.87) [5–13]	278.26 (54.89) [206–443]	228 (54.4) [144–341]
Controls	43.16 (6.31) [35.32–5.43]	-	-	-	-	237.85 (52.53) [177–314]	495.75 (137.95) [268–763]

We show characteristics of cosmonauts and controls, such as age and length of previous missions. We then report the duration of the current mission and the time when the MRIs were collected before (T1) and after the spaceflight (T2). We also add the interval between baseline and post-flight (Interval T1-T2), as well as the gap between post-flight and the follow-up (Interval T2-T3). Every measurement is reported with the average and standard deviation (in parenthesis).

We collected high-resolution T1-weighted magnetic resonance imaging (T1-w MRI) data on a 3 T MRI GE Discovery MR750 scanner equipped with a 16-channel receiver head coil located at the National Medical Research Treatment and Rehabilitation Center of the Ministry of Health of Russia in Moscow. Imaging parameters include a repetition time (TR) of 8 ms, echo time (TE) of 3 ms, flip angle (FA) of 12°, 1 mm isotropic voxel size, and a field-of-view (FOV) of 240 x 240 x 180 mm^3^.

### Individualized biophysical modeling

**Fig. 3:**
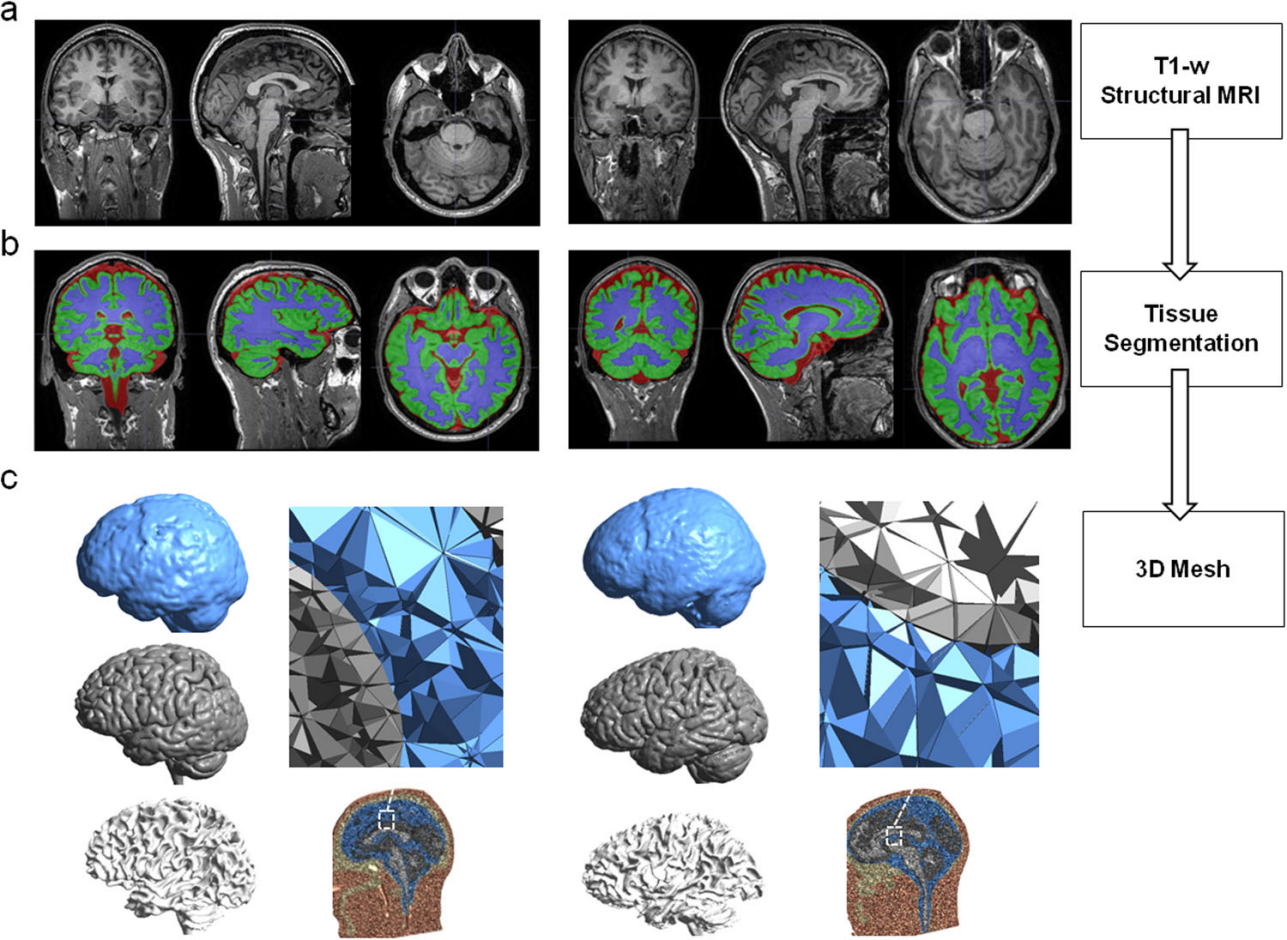
Image processing and mesh creation diagram. We show the pipeline of image processing to create a 3D mesh to run the simulations for two participants in the study as an example, one cosmonaut (left) and one control participant (right). **a** We collected the T1-weighted (T1-w) anatomical scan for each participant on three time-points (pre-flight, post-flight, and follow-up). In the figure, we show MRIs collected pre-flight for both participants. **b** We ran the *headreco* + *CAT* pipeline from SimNIBS^13^ to segment the T1 anatomical image into class types (skin, bone, CSF, eyes, gray matter, and white matter). For better visualization, we show segmentation of CSF (red), gray matter (green), and white matter (blue). **c** We then computed the head volume 3D meshes composed of tetrahedral elements (CSF: blue; gray matter: gray; white matter: white). Thanks to this process we can enter coordinates in MNI and the software will convert them to personal subject space to ensure accuracy in targeting.

MRI data quality assurance (sample homogeneity), preprocessing, and analysis were performed with CAT12 Toolbox (version 1727)^44^ (http://www.neuro.uni-jena.de/cat) within SPM12 (Wellcome Department of Cognitive Neurology, London, UK), using Matlab R2019a (Mathworks, Natick, MA, USA; see Fig. 3, panel a). The induced E-field of the TMS was computed in SimNIBS v3.2, an open-source simulation package that integrates segmentation of MRI scans, mesh generation, and FEM model E-field computations^13,14^. The software provides a realistic volume conductor head model, created as default in FEM generated using the individual T1-weighted image. The finite element head model for each participant was reconstructed by applying the *headreco* + *CAT* pipeline to the subject’s T1-weighted scans. In our simulation, we kept the isotropic conductivities given by default^14^, here provided for the main tissue volumes in S/m: gray matter: 0.275; white matter: 0.126; CSF: 1.654; bone/skull: 0.01; scalp/skin: 0.465. The final mesh, comprehensive of gray (GM) and white matter (WM), scalp tissue, bone, and cerebrospinal fluid, comprises ~200,000 nodes and 3.6 million tetrahedral elements (see ref. ^45^) for further modeling details) for each participant (see Fig. 3, panels b and c). Segmentations were carefully examined slice-by-slice to ensure proper tissue classifications.

### TMS simulations and targeting

E-field distribution was computed with SimNIBS^13^ model of the Magstim 70 mm figure-of-8 coil (P/N 9925-00, Magstim Co., Spring Gardens, Whitland, Carmarthenshire, UK). The coil has nine windings with outer and inner diameters of 8.8 and 5.2 cm, respectively^40^. The coil-to-scalp distance was set at 4 mm (by default in the software). As modeled in SimNIBS, the E-field input is in the form of dI/dt in units of A/us. The dI/dt is the speed of variation of the current through the coil. Its value depends on the coil model, stimulator model, and pulse intensity (for a detailed explanation see ref. ^46^). Coils have different inductances (*L* in micro-henrys). The stimulator output (% of the maximum capacitor’s charging voltage in the stimulator) will result in different max dI/dt values. The expression is d*I*/d*t*|Max = Vc/*L*, where Vc is the capacitor’s charging voltage and L is the inductance of the coil. For our simulations we assume to use a Magstim 200 stimulator (Vc|Max = 2800 V) and the inductance of the figure-8 70 mm coil is 16.35 uH. This would result in a max dI/dt value (at Max stimulator output, MSO) of 171.3 A/us. Assuming a Resting Motor Threshold (RMT) of around 40%, and knowing that variation with pulse is linear, we chose a dI/dt of 70 A/us (40.8% of the MSO 171.3 A/us).

**Fig. 4:**
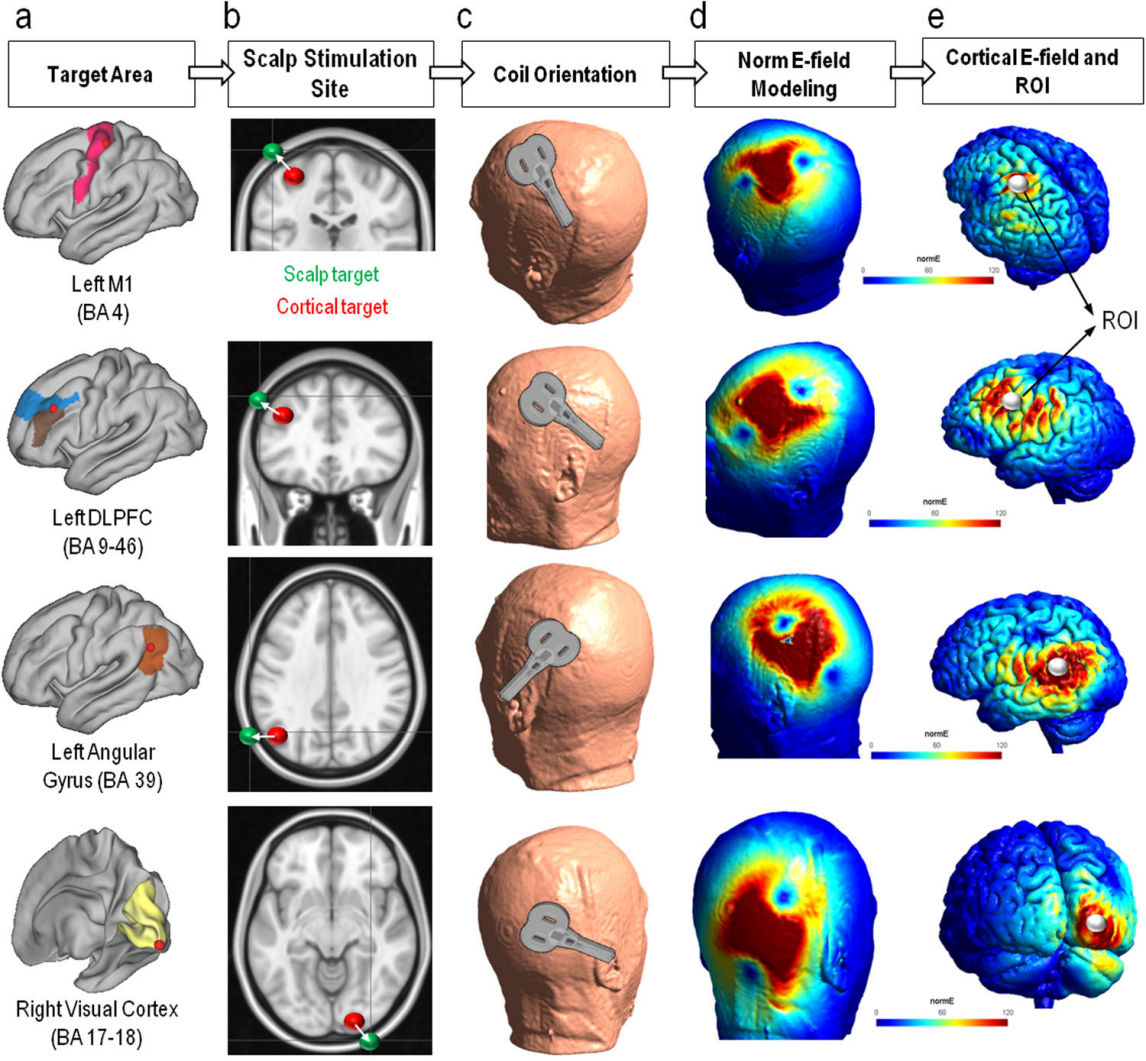
Process for TMS simulations and NormE value extraction. The figure shows the process used to identify the stimulation sites, TMS coil placement and orientation, and extraction of the resulting NormE. **a** We focused on four regions as potential stimulation targets for their relevance for neuromodulation protocols in space travelers: left primary motor cortex (M1), left dorsolateral prefrontal cortex (DLPFC), left angular gyrus, and right visual cortex. The set of coordinates for each cortical target was chosen from up-to-date literature (see main text for details). The panel shows the targets (red spheres) and the corresponding Brodmann Area (BA). **b** We created a set of spherical region-of-interest (ROI) centered in the coordinates (red spheres enlarged in the figure to allow visualization). The ROIs were overlapped on the MRI T1-w in an MNI standard brain and moved 90 degrees oblique toward the outside until reaching the scalp. We saved the position on the skin as scalp targets (green spheres). **c** We centered the coil in this new set of coordinates for stimulation sites on the scalp. The coil handle was then manually rotated to recreate the orientation normally employed in an experimental setting. In the main text, we also report the set of MNI coordinates for the corresponding orientation of the coil handle, in order to ensure the same precise orientation for each participant. All MNI coordinates were converted to subject space thanks to SimNIBS. **d** We then ran the simulation of TMS pulse computing the resulting NormE distribution for each participant, at each time-point (baseline, post-flight, follow-up) on the four stimulation sites. The resulting output from one of the cosmonauts enrolled in the study is shown in the panel. **e** To compute the current strength reached by the cortical areas we were interested in, the NormE was extracted in the gray matter of the area stimulated for each individual at each time-point. The ROI spheres previously created with coordinates in cortical targets are here presented in white and enlarged to allow visualization. We intersected them with the layer of gray matter within the TMS-generated electric field. The weighted average NormE in every tetrahedron of the mesh within the ROI was computed. The figure shows the NormE in V/m. Note = M1: primary motor cortex, DLPFC: dorsolateral prefrontal cortex, BA: Brodmann area, ROI: region-of-interest.

We then simulated the impact of TMS over four cortical targets potentially relevant for neuromodulation protocols in space missions^2^: left primary motor cortex (M1), left dorsolateral prefrontal cortex (DLPFC), left angular gyrus, and right visual cortex (V1/V2). To do so, we chose the coordinates for each stimulation site according to literature. The left M1 coordinates were taken from Mayka et al., a meta-analysis of 126 articles with the aim to locate different motor regions with high accuracy^47^. The coordinates were in Talairach *[x* = *–37, y* = *–21, z* = *58]*, and converted in MNI thanks to an online toolbox (https://bioimagesuiteweb.github.io/webapp/mni2tal.html) *[x* = *–37, y* = *–25, z* = *64]*. We chose the left DLPFC coordinates [*x* = *–40, y* = *31, z* = *34]* from Cho and Strafella, a study showing that repetitive TMS (rTMS) on this cortical target produced dopamine release in cingulate and orbitofrontal areas relevant for improving learning and depressive symptoms^48^. We extracted the coordinates for the stimulation on the left angular gyrus *[x* = *–48, y* = *–64, z* = *30]* from an fMRI-TMS study indicating it as the center of the region pivotal for episodic memory^49^. The coordinates corresponding right V1/V2 *[x* = *25, y* = *–92, z* = *–9]* were taken from Cocchi et al., a study of neuroimaging, non-invasive cortical stimulation, and computational modeling to investigate the visual cortex^50^. We kept these 4 sets of coordinates as our cortical targets (see Fig. 4, panel a).

When choosing a target, we can enter coordinates within the brain and SimNIBS will automatically project them to the closest skin surface. This can cause a shift of the stimulation site and differences among subjects due to variance in skull/brain structure. To overcome this, we created a set of ROI spheres with the center as the coordinates in cortical target areas and 3 mm diameter (red spheres in Fig. 4, panel b). We overlapped them on the T1-weighted in an MNI standard brain used as example dataset (Ernie) included in SimNIBS software^45^. All the sites’ coordinates were visually inspected on the T1-w images to confirm their spatial correspondence to the target gray matter regions. We then moved 90 degrees oblique toward the scalp until reaching it and saved the position on the skin as the new target. We saved these new sets of coordinates as stimulation sites on the scalp: left M1 *[x* = *–51, y* = *−25, z* = *81]*, left DLPFC *[x* = *–55, y* = *31, z* = *46]*, left angular gyrus *[x* = *–69, y* = *–65, z* = *32]*, and right visual cortex *[x* = *33, y* = *–108, z* = *–9]*. All the coordinates are presented in MNI space. For each participant, these MNI coordinates were converted to subject space in SimNIBS. This was our set of coordinates for stimulation sites on the scalp, corresponding to the center of the coil (green spheres in Fig. 4, panel b).

The coil handle was then manually moved on the SimNIBS GUI and rotated according to the specified orientation that is usually used in real-life setting. We added a set of MNI coordinates for the corresponding orientation of the coil handle, in order to ensure an accurate representation of the generated E-field: left M1 *[x* = –*41, y* = *–8, z* = *85]*, left DLPFC *[x* = *–44, y* = *44, z* = *56]*, left angular gyrus *[x* = *–57, y* = *–80, z* = *54]*, and right visual cortex *[x* = *14, y* = *–118, z* = *5]*. These coordinates were converted in subject space as well during the simulations (see Fig. 4, panel c).

The resulting NormE distribution for each participant, on any time-point (pre-flight, post-flight, follow-up) on the 4 stimulation sites is shown in Gmsh v4.7.1^51^. The output is a mesh with nodes scattered in space forming tetrahedra with defined electric fields (and current density fields) in each element (see Fig. 4, panel d). To investigate specific changes in current strength generated by TMS in our target areas, we extracted the mean value of the NormE field in the gray matter of the area stimulated for each individual at each time-point. We used the ROI spheres we previously created with coordinates in cortical relevant targets and masked them with the layer of gray matter within the mesh. SimNIBS will then get the center of the tetrahedral included in the GM layer inside the sphere and calculate the NormE in the ROI using a weighted average (see Fig. 4, panel e). The process starts from the final mesh created from the individual segmented MRI scan including ~3.6 million tetrahedral elements. SimNIBS computes the precise volume of every single tetrahedron within the chosen ROI. It will then calculate the NormE within every single tetrahedron. Finally, the average of the NormE in all tetrahedral elements is computed by weighting the electric field values based on the volume of each tetrahedron. The output is the average intensity as represented from NormE currently represented in V/m.

### Analysis of CSF volume

Automated segmentation of CSF volume was performed with Freesurfer software package Version 6.0 (http://surfer.nmr.mgh.harvard.edu/). Freesurfer volume-based subcortical segmentation pipeline has been extensively described in previous literature^52^. Briefly, Freesurfer uses a probabilistic atlas that is built by manually labeling a training dataset, which is then normalized to the MNI space to achieve a point-to-point correspondence between all the training subjects. The atlas provides the probability of each label at each voxel, the probability of each label given the classification of neighboring voxels (neighborhood function), and the probability distribution function of voxel intensities, modeled as a normal distribution, for each label at each voxel. The segmentation of a new image is achieved by normalizing the new subject to the common space and incorporating the subject-specific voxel intensities to find the optimal segmentation that maximizes the probability of observing the input data. We extracted CSF volume for each participant at each time-point. Freesurfer separately extracts the volume of each ventricle (left and right inferior lateral ventricles, left and right superior lateral ventricles, 3rd ventricle, and 4th ventricle). We added the volume of the ventricles (filled with CSF) to the peripheral CSF volume to compute the total volume of the CSF and use the output for the analysis. The values are presented in mm^3^.

### Statistical analysis

All data were analyzed using SPSS version 22 (SPSS Inc., Chicago, USA). We ran separate analyses for every variable (generated NormE in four target regions, total CSF volume). Changes in NormE were computed separately for each stimulation site with repeated measure two-way analysis of variance (ANOVA), with Time as a within-subjects factor (pre-flight vs. post-flight, pre-flight vs. follow-up, post-flight vs. follow-up) and Group as a between-subjects factor (2 groups: cosmonauts vs. control). We report the *p*-values for the interaction group vs. time. To be sure that significant differences in the control group over time would not confound the effects seen in cosmonauts, we performed one-way ANOVA with Time as a within-subjects factor (pre-flight vs. post-flight, pre-flight vs. follow-up, post-flight vs. follow-up) for each variable in this cohort alone.

Correlation analysis was then performed to assess associations between changes in NormE and modifications in brain anatomy. Specifically, we performed correlation analysis for delta (post–pre) in total CSF volume with the delta of NormE in each area. For every correlation, we added Group as a second independent variable. A *p*-value lower than 0.05 was considered statistically significant.

### TMS optimization

To show how the personalization of TMS parameters allows a more accurate stimulation, we provide solutions to resolve the difference in current strength between pre- and post-flight. The induced electric field distribution depends on multiple parameters such as intensity of the stimulation, location, and orientation of the TMS coil. For this example, we offer two potential solutions of personalization: (i) changing the intensity of the TMS pulse and running a new simulation; (ii) performing TMS optimization to find the best coil position and orientation. In both cases we ran these processes on post-flight scans, to account for the spaceflight-induced brain structural modifications.

For the first solution, we started by normalizing the difference in NormE reached in TMS simulations over M1 at post-flight compared to baseline (100*(Post–Pre)/Pre) in two cosmonauts. This is the change of the current strength in percentage after the space mission. Knowing that pulse intensity and current are linear, we re-ran the TMS simulations on post-flight data over M1, modifying the dl/dT by lowering the score we used before (70 A/us) by the percentage change. The resulting NormE is shown in Gmsh v4.7.1^51^ with an output range in V/m. After calculating the strength of the field in the ROI with the process previously described, we compared it to the result in TMS simulation M1 on the same post-flight MRI of the same participant.

The second solution was performed by implementing a SimNIBS function (*TMSoptimize*) that computes the best TMS coil position and orientation to stimulate a certain target^41^. We ran this analysis on the mesh of post-flight data for the same two cosmonauts with target in the left angular gyrus (cortical coordinates as above: *[x* = –*48, y* = *–64, z* = *30])*. The software starts by searching coil positions in a grid around the target and turning the coil at various angles for a total of 540 possible trials^41^. SimNIBS returns the position and orientation of the coil that induces the largest NormE at the target. At the end of the process, we compared the resulting NormE with the previous output in TMS stimulation over the same target on the post-flight MRI of the participant.

### Reporting summary

Further information on research design is available in the Nature Research Reporting Summary linked to this article.

## Supplementary information

Reporting Summary

## Data Availability

All data needed to evaluate the conclusions in the paper are present in the paper. Additional preprocessed data related to this paper may be requested by directly emailing the authors.
