# Supplementary material for: Optimizing transcranial magnetic stimulation for spaceflight applications
Source: NPJ Microgravity. 2023 Mar 28;9:26. doi: 10.1038/s41526-023-00249-4 (PMC10050431; doi:10.1038/s41526-023-00249-4)
Supplement: Supplementary file 1 — Reporting Summary [file 41526_2023_249_MOESM1_ESM.pdf]

nature portfolio

Corresponding author(s): Emiliano Santarnecchi

Last updated by author(s): Dec 1, 2022

Reporting Summary

Nature Portfolio wishes to improve the reproducibility of the work that we publish. This form provides structure for consistency and transparency in reporting. For further information on Nature Portfolio policies, see our Editorial Policies and the Editorial Policy Checklist.

Statistics

For all statistical analyses, confirm that the following items are present in the figure legend, table legend, main text, or Methods section.

n/a	Confirmed	
☐	☒	The exact sample size (n) for each experimental group/condition, given as a discrete number and unit of measurement
☐	☒	A statement on whether measurements were taken from distinct samples or whether the same sample was measured repeatedly
☐	☒	The statistical test(s) used AND whether they are one- or two-sided<br>*Only common tests should be described solely by name; describe more complex techniques in the Methods section.*
☐	☒	A description of all covariates tested
☐	☒	A description of any assumptions or corrections, such as tests of normality and adjustment for multiple comparisons
☐	☒	A full description of the statistical parameters including central tendency (e.g. means) or other basic estimates (e.g. regression coefficient) AND variation (e.g. standard deviation) or associated estimates of uncertainty (e.g. confidence intervals)
☐	☒	For null hypothesis testing, the test statistic (e.g. F, t, r) with confidence intervals, effect sizes, degrees of freedom and P value noted<br>*Give P values as exact values whenever suitable.*
☒	☐	For Bayesian analysis, information on the choice of priors and Markov chain Monte Carlo settings
☒	☐	For hierarchical and complex designs, identification of the appropriate level for tests and full reporting of outcomes
☐	☒	Estimates of effect sizes (e.g. Cohen's d, Pearson's r), indicating how they were calculated

Our web collection on statistics for biologists contains articles on many of the points above.

Software and code

Policy information about availability of computer code

Data collection	No custom codes were used for the data collection.
Data analysis	Data analysis was ran with existing code.

For manuscripts utilizing custom algorithms or software that are central to the research but not yet described in published literature, software must be made available to editors and reviewers. We strongly encourage code deposition in a community repository (e.g. GitHub). See the Nature Portfolio guidelines for submitting code & software for further information.

Data

Policy information about availability of data

All manuscripts must include a data availability statement. This statement should provide the following information, where applicable:

- Accession codes, unique identifiers, or web links for publicly available datasets
- A description of any restrictions on data availability
- For clinical datasets or third party data, please ensure that the statement adheres to our policy

All data needed to evaluate the conclusions in the paper are present in the paper. Additional preprocessed data related to this paper may be requested by directly emailing the authors.

Human research participants

Policy information about studies involving human research participants and Sex and Gender in Research.

Reporting on sex and gender	Only male human subjects took part in our study, which is the result of the available test subjects during the course of our study and not an active choice. Data analyses were performed on Russian cosmonauts who had scheduled flights to the International Space Station according to the Russian space agency Roscosmos. The cosmonauts took part in our study on a voluntary basis and no participants were denied participation.
Population characteristics	The average age of the cosmonaut group was 46.06 years. All cosmonauts were male, which was purely determined by the ISS flight schedule. All cosmonauts engaged in ISS missions with an average mission duration of 184.46 days.
Recruitment	Cosmonauts were recruited through Roscosmos. Roscosmos will propose to each cosmonaut going to the ISS to participate in our study. The cosmonauts then participate on a voluntary basis. All cosmonauts have the chance to participate in our study. If cosmonauts do not participate or quit the study along the way, this is beyond the influence of the researchers/authors, and thus no selection bias is present in our data pool.
Ethics oversight	The study was approved by the Institutional Review Board of the Antwerp University Hospital (13/38/357), the Committee of Biomedicine Ethics of the Institute of Biomedical Problems of the Russian Academy of Science, and the Human Research Multilateral Review Board. All participants provided a signed informed consent and all investigations were performed in accordance with the principles listed in the Declaration of Helsinki and its amendments.

Note that full information on the approval of the study protocol must also be provided in the manuscript.

Field-specific reporting

Please select the one below that is the best fit for your research. If you are not sure, read the appropriate sections before making your selection.

☒ Life sciences ☐ Behavioural & social sciences ☐ Ecological, evolutionary & environmental sciences

For a reference copy of the document with all sections, see nature.com/documents/nr-reporting-summary-flat.pdf

Life sciences study design

All studies must disclose on these points even when the disclosure is negative.

Sample size	15 cosmonauts were included in the study, who were tested two times, (preflight and postflight). 11 of these cosmonauts were also tested a third time (follow-up). The data drop-out was due to voluntarily quitting the study for 3 cosmonauts, and a pending followup measurement for 1 cosmonaut. 14 control subjects were also included in the study, who were tested twice with a similar time interval as the duration of the space mission. Sample size was determined by having at least 10 participants scanned at each of the three time points.
Data exclusions	Only participants who were tested at least at preflight and postflight were included. No data were excluded after this initial selection criterion.
Replication	Due to the logistic difficulties in acquiring data from cosmonauts and considering the long time span to replicate the current study, we were not able to perform replication analyses.
Randomization	Groups were predetermined by having cosmonauts who engaged in missions to the International Space Station in one group, and control participants who stayed on Earth in another control group.
Blinding	No blinding procedure was implemented in this study. The results of this study were not dependent on subjective assessments.

Reporting for specific materials, systems and methods

We require information from authors about some types of materials, experimental systems and methods used in many studies. Here, indicate whether each material, system or method listed is relevant to your study. If you are not sure if a list item applies to your research, read the appropriate section before selecting a response.

Materials & experimental systems

n/a	Involved in the study
☒	☐ Antibodies
☒	☐ Eukaryotic cell lines
☒	☐ Palaeontology and archaeology
☒	☐ Animals and other organisms
☒	☐ Clinical data
☒	☐ Dual use research of concern

Methods

n/a	Involved in the study
☒	☐ ChIP-seq
☒	☐ Flow cytometry
☐	☒ MRI-based neuroimaging

Magnetic resonance imaging

Experimental design

Design type: N/A

Design specifications: N/A

Behavioral performance measures: N/A

Acquisition

Imaging type(s): structural

Field strength: 3T

Sequence & imaging parameters: 3D Fast spoiled gradient recalled acquisition (FSPGR): TE=3ms; TR=8ms; flip angle=12°, FOV=240x240x180mm3; voxel sizes: 1mm isotropic

Area of acquisition: whole brain

Diffusion MRI: ☐ Used ☒ Not used

Preprocessing

Preprocessing software: Freesurfer software package Version 6.0

Normalization: Non-linear normalization of T1w scans based on volume-based subcortical segmentation pipeline

Normalization template: MNI

Noise and artifact removal: Inhomogeneities and noise are removed and intensities are globally normalized.

Volume censoring: N/A

Statistical modeling & inference

Model type and settings: repeated measure two-way analysis of variance (ANOVA); and linear regression.

Effect(s) tested: Cosmonaut group vs. Control group, in NormE and CSF changes pre vs postflight.
Correlation analyses between MRI-based volume changes and changes in modeled biophysical parameters

Specify type of analysis: ☐ Whole brain ☒ ROI-based ☐ Both

Anatomical location(s): Total cerebrospinal fluid volume (CSF).

Statistic type for inference (See Eklund et al. 2016): N/A

Correction: N/A

Models & analysis

n/a	Involved in the study
☒	☐ Functional and/or effective connectivity
☒	☐ Graph analysis
☒	☐ Multivariate modeling or predictive analysis
